# Notes and News

**Published:** 1905-12-23

**Authors:** 


### Notes and News.

Mr. Albert Bowden, who died on October 15, bequeathed £5,000 to the Stockport Infirmary, and £4,000 to the British and American Orphanage in Paris.

A lady has offered to give £1,000 towards the £3,000 debt to the bankers incurred by the Royal Hospital for Incurables, Putney, if the other £2,000 are obtained by the new year.

One result of motoring and motor accidents is shown by the gift of a revolving open-air shelter to the Stephen Cottage Hospital, Dufftown, by Mr. Leyland, a motorist who met with an accident and was treated at this hospital.

The Waterloo Hospital for Women and Children, the Bethlem Hospital for Mental Diseases, and the General Lying-in Hospital have been affiliated to the London School of Clinical Medicine. The London School of Clinical Medicine is thus enabled to provide its post-graduates with facilities for practical study in every branch of medicine and surgery.

Mrs. Eliza Eyre, of King's Hill, Dursley, Gloucester, who died on September 24, bequeathed £2,000 to St. George's Hospital, £2,000 to Guy's Hospital, £2,000 to the Royal Sea-Bathing Hospital at Margate, £2,000 to the Gloucester General Infirmary, £1,000 to the London Hospital, £1,000 to the Bristol Infirmary, £1,000 to the Bristol General Hospital, £500 to the Gloucester Free Children's Hospital, £500 to the West of England Sanatorium, Weston-super-Mare, and £500 to the Convalescent Home, Walton-by-Clevedon.

By the will of the late Mr. Alfred J. Bishop, of Newark-upon-Trent, the Newark Hospital and Dispensary and the Nottingham and Nottinghamshire Association for the Prevention of Consumption will each benefit to the extent of £250.

The London County Council have adopted the recommendation of their General Purposes Committee with regard to an ambulance service for London. The scheme, which is looked upon as an experimental one, includes (1) the provision of two ambulance stations, one on the north side and the other on the south side of the river; (2) motor ambulances and wheeled litters; (3) a method of giving calls by means of street call-posts fitted with telephones. The expenditure involved in the scheme for the first year is estimated at £5,200, including £2,200 for the initial outlay.

In giving evidence before the Royal Commission on the care and control of the feeble-minded, the Duchess of Bedford urged that if new institutions devoted to the segregation of female degenerates were to be established as a result of the Commission's report, educated women should be employed as superintendents. In such institutions abroad a woman of superior education is usually appointed as superintendent, and this course has been shown by experience to be of the greatest benefit to the inmates. At present there is too great a tendency to maintain artificial conditions of comfort out of all proportion to the social status of the inmates, while at the same time their mental and moral culture is to a large extent neglected.

It was announced on Tuesday by General Booth that Mr. George Herring has placed at the disposal of the Salvation Army a sum of £100,000 for the carrying out of a scheme of home colonisation of the unemployed. A portion of this sum is at once to be expended in an initiatory experiment, and the remainder is to be furnished as required. The scheme involves the provision for each settler of five acres of suitable land. But our main interest centres in the fact that the whole amount advanced by Mr. Herring is ultimately to be repaid by the Salvation Army to King Edward's Hospital Fund in annual instalments of £4,000. Mr. Herring has thus achieved the twofold purpose of placing at the disposal of the Salvation Army the means required to practically test a promising scheme for providing work for the deserving poor, and of making a very substantial and very welcome addition to his splendid benefactions to the London hospitals. This new act of generosity may appropriately be regarded as the Christmas gift of Mr. Herring to the charities.

